# Using the community-based health planning and services program to promote skilled delivery in rural Ghana: socio-demographic factors that influence women utilization of skilled attendants at birth in Northern Ghana

**DOI:** 10.1186/1471-2458-14-344

**Published:** 2014-04-10

**Authors:** Evelyn Sakeah, Henry V Doctor, Lois McCloskey, Judith Bernstein, Kojo Yeboah-Antwi, Samuel Mills

**Affiliations:** 1 Social Science Unit, Navrongo Health Research Centre, Upper East Region, Navrongo, Ghana; 2 Integrated Programme and Oversight Branch, Division for Operations, United Nations Office on Drugs and Crime, Abuja, Nigeria; 3 Community Sciences Department, Boston University School of Public Health, Boston, MA, USA; 4 International Health Department, Boston University School of Public Health, Boston, MA, USA; 5 Health, Nutrition, and Population, Human Development Network, The World Bank, Washington DC, USA

**Keywords:** Community-based service delivery, Ghana, Maternal mortality, Women service utilization, Skilled attendants at birth

## Abstract

**Background:**

The burden of maternal mortality in sub-Saharan Africa is enormous. In Ghana the maternal mortality ratio was 350 per 100,000 live births in 2010. Skilled birth attendance has been shown to reduce maternal deaths and disabilities, yet in 2010 only 68% of mothers in Ghana gave birth with skilled birth attendants. In 2005, the Ghana Health Service piloted an enhancement of its Community-Based Health Planning and Services (CHPS) program, training Community Health Officers (CHOs) as midwives, to address the gap in skilled attendance in rural Upper East Region (UER). The study determined the extent to which CHO-midwives skilled delivery program achieved its desired outcomes in UER among birthing women.

**Methods:**

We conducted a cross-sectional household survey with women who had ever given birth in the three years prior to the survey. We employed a two stage sampling techniques: In the first stage we proportionally selected enumeration areas, and the second stage involved random selection of households. In each household, where there is more than one woman with a child within the age limit, we interviewed the woman with the youngest child. We collected data on awareness of the program, use of the services and factors that are associated with skilled attendants at birth.

**Results:**

A total of 407 households/women were interviewed. Eighty three percent of respondents knew that CHO-midwives provided delivery services in CHPS zones. Seventy nine percent of the deliveries were with skilled attendants; and over half of these skilled births (42% of total) were by CHO-midwives. Multivariate analyses showed that women of the Nankana ethnic group and those with uneducated husbands were less likely to access skilled attendants at birth in rural settings.

**Conclusions:**

The implementation of the CHO-midwife program in UER appeared to have contributed to expanded skilled delivery care access and utilization for rural women. However, women of the Nankana ethnic group and uneducated men must be targeted with health education to improve women utilizing skilled delivery services in rural communities of the region.

## Background

High maternal mortality in developing countries remains a challenge. Maternal death is “the death of a woman while pregnant or within 42 days of termination of pregnancy from any cause related to or aggravated by the pregnancy or its management, but not from accidental or incidental cause” [1].

According to WHO, UNICEF, UNFPA, and The World Bank, sub-Saharan Africa accounted for 56% of the global total estimated maternal deaths of 287,000 in 2010 [2,3]. Additionally, about 60 million women worldwide have poor reproductive health or serious pregnancy-related illnesses or disability including fistulae, uterine prolapse, and infertility [4]. Ghana has a high maternal mortality ratio (MM Ratio) of 350 maternal deaths per 100,000 live births [2].

Access to and quality skilled attendants at birth and emergency obstetric care [5] has been shown to be effective in reducing maternal mortality [4]. Skilled birth attendance is defined as “the availability of health professionals with midwifery skills (doctors, nurses or midwives) to promote utilization, conduct normal deliveries and provide first aid, the enabling environment of health policy and system, drugs, equipment, supplies, and transportation, knowledge and skills to refer from one level of skilled attendance to another and the demand for skilled care by community as evidenced by utilization” [6,7]. One of the key indicators for monitoring progress towards the Millennium Development Goal (MDG) 5 is the proportion of births by skilled attendant. In Ghana, two-thirds of women deliver with the assistance of skilled attendants, but skilled delivery is higher in urban (88%) than in rural areas (54%) [8]. Nevertheless, the level of skilled delivery in the rural areas of UER (67%) is similar to that of urban areas in Ghana [8].

The Community-Based Health Planning and Services (CHPS) program was established in 2000 to improve access and quality of health care and family planning services in Ghana. The CHPS Initiative emanated from the Navrongo experiment known as the Community Health and Family Planning (CHFP) project designed as a community-based model for providing integrated health services to rural communities. The experiences and lessons of the ‘Navrongo Experiment’ serve as the basis for the establishment of the CHPS program. The CHPS program is implemented by the Ghana Health Service (GHS), but with substantial contribution from the local communities with the provision of land and labor for building CHPS compounds [9]. Community volunteers undertake health education and management of minor ailments [9].

The GHS has trained a cadre of health care providers known as Community Health Officers (CHOs) to provide skilled attendance at delivery to women in rural areas through the CHPS program. A CHO-midwife is an auxiliary nurse, who is also trained in midwifery to provide basic health services including skilled delivery care in rural areas [9]. If the CHPS program is effective in promoting skilled attendants at delivery it may address the geographical, cultural, socio-economic and ethnicity factors that limit access to maternity care by making health services *available* through close proximity to rural families, socially *accessible* through wide community participation, and *affordable* through free delivery system.

Against this background, the main objectives of this study are to ascertain women awareness of the skilled delivery program; determine the percentage of women who have delivered with the CHO-midwives, and examine factors that influence women decision to have skilled attendants at birth in rural areas such as geography and accessibility [10-12], socio-economic factors [11-15] and traditional beliefs and ethnicity factors [10-20].

## Methods

### Study setting

The study was conducted in the Kassena-Nankana East (KNE), Kassena-Nankana West (KNW), and Bongo Districts of the Upper-East region (UER) of Ghana. The UER, in northern Ghana, is one of the two regions in Ghana which is most remote from Accra, the capital. According to the 2010 census, the total population of the region is 1,046,545. The KNE district had an estimated population of 109,944 whereas the KNW district, newly carved out of the Kassena-Nankana District in UER, had an estimated population of 70,667 in 2012. Bongo district’s 2010 estimated census population was 84,545. KNE and KNW are predominately Kassenas and Nankanas and the Bulsas as a minority group in KNE. In the Bongo district, the people are mostly Frafras [21].

The people of UER share similar social and cultural practices such as funeral and widowhood rites, festivals, marriage customs and child naming [17]. Households are grouped into extended family units or compounds, each headed by a male. Lineage, customs, religious practices, marriage patterns, and other social characteristics of the population are traditional, but social changes such as construction of roads, schools and hospitals, among other things, are taking place [17].

### Ethics approval

We obtained ethical approval for this study from the Navrongo Health Research Centre, Ghana and the Boston University (BU) Institutional Review Boards (BU IRB reference number H-31245).

### Study design and methods

We conducted a household survey with women who had ever given birth in the three years prior to the study to ascertain their awareness of the CHPS program, use of the services, and determine factors that are associated with skilled attendants at birth. The predictor variables included ethnicity, husband’s education, woman’s education, age group, employment status, type of employment, religion, distance to health facility and number of children.

### Sampling and sample size

The sample size was calculated based on a proportion of deliveries supervised by trained professionals of 50% in the UER with annual births of 8,918 in the three districts and 95% confidence interval as well as a corresponding p < 0.05 for significance. We used the formula sample size *n* = [DEFF*Np (1-p)]/[(d^2^/Z^2^_1-α/2_*(N-1) + p*(1-p)] [22] and this gave us the sample size of 369 women. Assuming a refusal rate of 10%, the total sample size for the three districts was 407. In each district, women were included in the study based on the proportion of deliveries in that district.

A two-stage sampling method was employed. The primary sampling unit was the enumeration area (EA), defined as the geographic area canvassed by one census representative. The EAs ranged from 96–187 in the three districts. Sampling EAs and households was based on the assumptions that: (1) CHO-midwives were working in most of these EAs in each of the three districts and (2) there is homogeneity in receipt of CHO-midwives services across the EAs.

A CHO-midwife supervises or covers at least 24 EAs in a district. The three districts were included because they were the only districts having CHO-midwives providing skilled delivery services in CHPS zones. In the first stage, 10 EAs were selected proportional to the size of the EAs with at least one CHO-midwife working. The research team obtained a list of all the compounds and households in the EAs with women who had given birth in three years prior to the survey by visiting households and compiling a comprehensive list of women in compounds and households with children under five years. In the second stage, we selected households randomly from the compound and household listing developed and the interviewers visited each randomly selected household and interviewed the woman with a child within the age limit. In each household, where there is more than one woman with a child within the age limit, we interviewed the woman with the youngest child. A total of 1,300 women were on the compound and household list compiled for the study.

### Data collection

The data was collected using a structured questionnaire from January 13, 2012 to May 31, 2012. The questionnaire had sections on women’s social and demographic characteristics, knowledge and utilization of skilled delivery services, reasons for use or non-use of skilled delivery services, and decision-making on place of birth. Validated questions used in similar surveys were adopted wherever possible. The research team identified and selected field supervisors and fieldworkers who are literate for the survey data collection. The training of field supervisors and fieldworkers consisted of lectures, role playing and pretesting. They were instructed on how to number the questionnaire, and check for consistency of responses, among others.

We pre-tested the questionnaire in selected communities in the three districts which were not part of the study. The interviews offered the fieldworkers the opportunity to practice interviewing techniques, and the questionnaire was further revised based on the pre-test. In order to ensure that data collected was of good quality, trained supervisors, and research team closely monitored and supervised the fieldworkers and checked the consistency of the responses.

### Data analyses

Descriptive statistics (frequencies and percentages) were used and where appropriate chi-square test was used to test for group differences. All p-values were two-tailed, and a value of p < 0.05 was considered statistically significant. To adjust for multiple determinants of women’s decision to have skilled attendants at birth, logistic multivariate regression was performed using STATA version 11. The outcome variable was women’s utilization of skilled attendants at birth (yes, no), and the explanatory variables included ethnicity, husband’s education, women’s education, and employment status, type of employment, religious affiliation, age group, number of children and distance to health facility. We adjusted for community effect in the analysis, but this was not found to be significant (P = 0.44).

## Results

### Background characteristics

A total of 407 women were interviewed yielding a response rate 100%: 165 women were from Bongo, and 121 women each from the KNE and KNW. Of all the respondents, 44% were Frafras, 30% were Nankanas, and 24% were Kassenas. About two-thirds (64%) of the respondents were Christians, 28% practiced traditional religion, and 9% were Muslims (Table 1). Nearly half of the women have had some formal education (mostly primary level). Similarly, nearly half of the husbands have had education but their level of education was higher compared with the women.

**Table 1:** Socio-demographic characteristics of female respondents in the Kassena-Nankana East and West Districts and the Bongo Districts of northern Ghana, 2012

**Characteristics**	**Kassena-Nankana East**	**Kassena-Nankana West**	**Bongo**	**All districts**
	**N (%) 121 (29.7)**	**N (%) 121 (29.7)**	**N (%) 165 (40.5)**	**N (%) 407 (100.0)**
**Age group**				
15-24	43 (35.5)	32 (26.4)	56 (33.9)	131 (33.3)
25-49	21 (17.3)	31 (26.6)	41 (24.8)	96 (24.4)
30-39	42 (34.7)	44 (36.4)	50 (30.3)	136 (34.5)
40-49	15 (12.4)	14 (11.6)	18 (10.9)	31 (7.9)
**Religion**				
Traditional	32 (26.4)	31 (25.6)	50 (30.3)	113 (27.8)
Christianity	81 (66.9)	84 (70.2)	93 (56.3)	259 (63.6)
Islam	8 (6.6)	6 (4.9)	22 (13.5)	35 (8.6)
**Ethnicity**				
Kassena	34 (28.9)	67 (55.4)	1 (0.6)	97 (23.8)
Nankana	82 (67.8)	40 (33.1)	0 (0.0)	123 (30.2)
Frafra	5 (4.1)	10 (8.3)	163 (98.8)	177 (43.5)
Other	2 (1.7)	4 (3.3)	1 (0.6)	7 (1.7)
**Marital status**				
Single	12 (9.9)	5 (4.1)	12 (7.3)	29 (7.1)
Living together	2 (1.7)	4 (3.3)	0 (0.0)	6 (1.5)
Widowed	1 (0.8)	3 (3.3)	7 (4.2)	11 (2.7)
Married	106 (87.6)	109 (90.1)	146 (88.5)	361 (88.7)
**Ever attended school**				
Yes	63 (52.1)	63 (52.1)	71 (43.0)	197 (48.4)
No	58 (47.9)	58 (47.9)	94 (56.9)	210 (51.6)
**Level of women’s education**				
Primary	38 (60.3)	35 (55.6)	43 (60.5)	116 (58.9)
Middle/Junior High School	22 (34.9)	22 (34.9)	25 (35.2)	69 (35.2)
Secondary	3 (4.8)	5 (7.9)	3 (4.2)	11 (5.6)
Tertiary	0 (0.0)	1 (1.5)	0 (0.0)	1 (0.5)
**Husband’s education**				
Yes	53 (49.1)	55 (30.6)	70 (39.6)	178 (48.8)
No	52 (48.1)	55 (30.6)	73 (40.6)	180 (49.3)
Don’t know	3 (2.8)	1 (14.3)	3 (42.9)	7 (1.9)
**Level of husband’s education**				
Primary	34 (64.2)	25 (45.5)	41 (58.6)	100 (56.2)
Middle/Junior High School	8 (15.1)	18 (45.0)	14 (20.0)	40 (22.5)
Secondary	11 (20.8)	12 (32.4)	14 (20.0)	37 (20.8)
Tertiary	0 (0.0)	0 (0.0)	1 (1.4)	1 (0.6)
**Number of children alive**				
1	33 (27.3)	31 (25.6)	39 (23.6)	103 (25.3)
2	24 (19.8)	14 (11.6)	39 (23.6)	77 (18.9)
3	18 (14.9)	25 (20.7)	42 (25.5)	85 (20.9)
4	23 (19.0)	27 (22.3)	28 (16.9)	78 (19.2)
5	16 (13.2)	17 (14.0)	14 (8.5)	47 (11.4)
6	7 (5.8)	7 (5.8)	3 (1.8)	17 (4.2)
**Currently employed**				
Yes	112 (92.6)	119 (98.3)	112 (67.9)	343 (84.3)
No	9 (7.4)	2 (1.7)	53 (32.1)	64 (15.7)
**Type of employment**				
Housewife	22 (19.6)	9 (7.6)	18 (16.1)	49 (14.4)
Farmer	48 (42.9)	58 (48.7)	54 (48.2)	160 (46.6)
Trader	27 (24.1)	33 (27.7)	29 (25.9)	89 (25.7)
Civil Servant	0 (0.0)	3 (2.5)	2 (1.8)	5 (2.6)
Others	15 (13.4)	16 (13.4)	9 (8.0)	40 (11.7)
**Distance to health facility**				
Less than 30 minutes	73 (60.3)	74 (61.2)	58 (35.2)	205 (50.4)
30 mins – 2 hrs	45 (37.2)	29 (23.9)	105 (63.6)	179 (44.0)
2 hrs – 4 hrs	3 (2.5)	18 (14.9)	2 (1.2)	22 (5.4)

Of the 407 respondents, 93% had heard of the CHOs working in their vicinity. When respondents were asked who they believed provided skilled delivery services in their community, most respondents (83%) reported CHO-midwives; 10% mentioned midwives/nurses, doctors in the health centers/clinics, and district hospitals; 5% reported the TBAs provide delivery services in their communities; and 2% mentioned the CHOs.

### Deliveries supervised by different professionals

**Figure 1:**
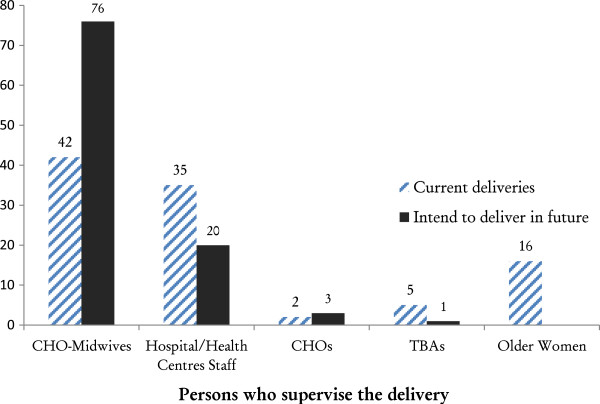
Percentage distribution of female respondents by person who assisted with past delivery and person preferred to assist with their delivery in future in the Upper East Region, 2012.

Figure 1 showed the percentage of women who delivered with different practitioners in the three districts. Nearly 80% of women delivered with the assistance of skilled birth attendants: 42% delivered with CHO-midwives; 35% with doctors or midwives at health facilities; 2% with CHOs, and 21% with TBAs or other older women. Regarding future deliveries, 76% of the women indicated preference to deliver with a CHO-midwife, while 20% intended to deliver with health personnel at the hospital or health center.

When asked about the persons involved with the decision-making on the place of the last delivery, 78% of respondents said they made the decision themselves, one-third noted that health professionals were involved in making the decision, and a similar proportion said that their husbands were involved with the decision making process. Further, 13% noted that their mothers-in-law played a role in the decision-making. Most respondents mentioned precipitous labor as the main reason for their inability to deliver with a CHO-midwife or in a health facility.

### Bivariate outcomes on women use of skilled attendants at birth

Table 2 presents bivariate results of use of health professional at delivery by selected characteristics. Without controlling for other socio-demographic factors, the Nankanas were less likely to be supervised by skilled attendants at birth. Further, women who were educated or married to educated husbands were more likely to have delivered with skilled birth attendants. Women who had one child were more likely to seek skilled care at birth than women with four or more children. However, employment status, religious affiliation, marital status, women and husband’s level of education and distance to health facility were not associated with women’s use of skilled professionals at birth.

**Table 2:** Bivariate analysis of women’s use of skilled attendants at birth, by selected characteristics (n = 407), Upper East Region, 2012

**Characteristics**	**Unadjusted odds ratio (95% confidence interval)**
**Ethnicity**	
Kassena (r)	**1.00**
Nankana	**0.23 (0.13-0.46)**
Frafra	1.09 (0.52-2.27)
Other	0.93 (0.10-8.35)
**Husband’s education**	
None (r)	**1.00**
Some formal education	**2.68 (1.57-4.58)**
**Husband’s level of education**	
None (r)	**1.00**
Primary	1.38 (0.47-3.99)
Middle/Junior High School	1.83 (0.50-6.69)
Secondary/Senior High School	2.40 (0.66-8.70)
**Woman’s education**	
None (r)	**1.00**
Some formal education	**1.78 (1.09-2.91)**
**Woman’s level of education**	
None (r)	**1.00**
Primary	1.46 (0.84-2.56)
Middle/Junior High School	**2.31 (1.07-4.96)**
Secondary/Senior High School	3.81 (0.48-30.1)
**Age group**	
15-24 (r)	**1.00**
25-29	0.62 (0.32-1.19)
30-39	0.61 (0.33-1.11)
40-49	0.90 (0.31-2.61)
**Employment status**	
No (r)	**1.00**
Yes	0.49 (0.22-1.06)
**Type of employment**	
Housewife (r)	**1.00**
Farmer	0.54 (0.28-1.04)
Trader	1.49 (0.73-3.04)
**Marital status**	
Single	0.85 (0.14-5.03)
Married	0.82 (0.17-3.89)
Widowed (r)	**1.00**
**Religion**	
Traditional	0.39 (0.14-1.08)
Christianity	0.75 (0.28-2.04)
Islam (r)	**1.00**
**Distance (in minutes)**	
Less than 30 minutes (r)	**1.00**
More than 30 minutes	1.01 (0.79-1.28)
**Number of children alive**	
1	**2.06 (1.04-4.06)**
2	1.15 (0.60-2.23)
3	1.14 (0.60-2.15)
4 or more (r)	**1.00**

Notes: Bolded values are significant at p < 0.05; r – reference category.

### Multivariate outcomes on women use of skilled attendants at birth

Table 3 presents the findings of the multivariate analysis. When we adjusted for other socio-demographic factors such as religion, employment status, age group, and number of children, the Nankanas were less likely to have had skilled attendants at birth compared with the Kassenas. Husband’s education and use of skilled delivery at birth was statistically significant association. However, there was no statistically significant association between the other variables noted above and skilled delivery at birth.

**Table 3:** Multivariate analysis of women’s use of skilled attendants at birth, by selected characteristics (n = 345), Upper East Region, 2012

**Characteristics**	**Adjusted odds ratio (95% conf. interval)**
**Ethnicity**	
Kassena (r)	**1.00**
Nankana	**0.24 (0.11-0.54)**
Frafra	1.46 (0.60-3.54)
Other	0.44 (0.08-2.53)
**Husband’s education**	
Not educated (r)	**1.00**
Some formal education	**2.17 (1.12-4.19)**
**Woman’s education**	
Not educated (r)	**1.00**
Some formal education	1.34 (0.69-2.58)
**Employed**	
No (r)	**1.00**
Yes	1.27 (0.47-3.42)
**Religion**	
Muslim (r)	**1.00**
Traditional	1.12 (0.59-2.23)
Christian	1.79 (0.53-6.08)
**Age group**	
15-24 (r)	**1.00**
25-29	0.59 (0.27-1.20)
30-39	0.68 (0.25-1.83)
40-49	0.77 (0.20-3.01)
**Distance**	
Less than 30 minutes (r)	**1.00**
More than 30 minutes	0.82 (0.45-1.41)
**Number of children**	
1	0.93 (0.33-2.62)
2	0.63 (0.25-1.59)
3	0.80 (0.37-1.80)
4 or more (r)	**1.00**

Note: Bolded values are significant at p < 0.05; r – reference category.

## Discussion

The findings revealed that majority of women have had skilled delivery three years prior to this study in CHPS zones where there was a CHO-midwife and many of the women preferred to be supervised by skilled attendants during birth in future. Multivariate analyses showed that women of the Nankana tribe and those with uneducated husbands were less likely to access skilled attendants at birth in rural settings.

Over four-fifths of women knew of the presence of a community health officer (CHO) in the community or that CHO-midwives provided skilled delivery services in the communities. Further, nearly 80% of the women sought skilled delivery care during their last birth and that could partly be attributed to the presence of the CHOs and CHO-midwives in the study area. Almost all the women preferred to deliver with a skilled attendant in future (76% with CHO-midwives) which demonstrated the positive influence of the presence of CHO-midwives in rural areas. Health education in these rural communities coupled with the availability of CHO-midwives and access to health facilities could have contributed to women use of skilled attendants. However, there is still the need for more sensitization since 21% of the women reported that they delivered with older women and TBAs in the communities.

In the multivariate analysis, husband’s education also showed statistically significant association with skilled delivery at birth. Husband’s decisions are important when it comes to who assisted with the woman’s delivery. In our study, husbands were not necessarily the sole decision-makers, yet they still played an important role in the decisions surrounding birth. Husbands are mostly family heads and key deciders on their families’ welfare and that power to take decisions extends to maternity care. Mills and Bertrand reported that among the Kassena-Nankana, decisions on place of delivery was the exclusive duty of the compound head, who is usually the senior male in the family or the husband of the woman or older women [11]. Almost all the women reported that they would deliver in a health facility in the future. That meant that if the skilled delivery program is well implemented in CHPS zones, every woman would be supervised by a skilled attendant at birth and that would guarantee the safety of women during delivery and accelerate attainment of MDG 5.

The Nankanas were less likely to have skilled delivery at birth compared with the Kassenas after controlling for other socio-demographic factors. There was no significant effect of the Frafra and ‘other’ ethnic groups on skilled attendants at birth. These findings are consistent with results from previous studies that revealed the significant role ethnicity plays in influencing women’s decision to seek skilled care at birth [12,13].

The Nankanas are largely traditionalists compared with the Kassenas who mostly practice Christianity. These ethnic differences are, partly related to the way Christianity was first introduced among the Kassenas because the early missionaries had few problems with their language (Kasem). This resulted in the Kassena communities experiencing social changes that included building of schools and hospital/clinics that were driven by Christian doctrines more than was the case in the Nankana areas [23].

We had a 100% response rate and achieving universal response rate in these three districts was possible since they are longstanding research sites for the Ghana Health Service and the Demographic and Health Surveys.

### Study limitations

The study is focused on skilled delivery program within the context of the CHPS Initiative and might not be generalizable to other contexts because of the uniqueness of the design and implementation of the CHPS program in Ghana. However, policy makers and program implementers in developing countries could still adapt the strategies to promote community-based programs, particularly the skilled delivery program, in rural areas. Additionally, recall bias could have been a limitation to the study because some participants might not have fully remembered past events about delivery care. However, using a 3-year recall period helped reduce the memory lapses that resulted from this study because it was relatively a shorter period for people to recall events such as place of delivery of last child. Another limitation of the survey research was the absence of baseline data to evaluate the before and after the implementation of the skilled delivery program to determine the increase in skilled deliveries due to the roll-out of the program in CHPS zones. We also lacked a comparison community to determine whether the skilled delivery program in CHPS zones actually contributed to improved skilled deliveries in rural settings.

## Conclusions and recommendations

The CHO-midwives activities have gained grounds in rural areas of the UER and the midwives have supervised the highest number of deliveries in the CHPS zones in the last three years. However, older women and some TBAs still supervise deliveries in rural communities and ethnicity and husband’s education played a role in women choice of place of delivery and precipitous labor contribute to unskilled deliveries in rural communities. Notably, majority of women preferred to deliver with the CHO-midwives in future.

In order to address sudden births—that usually occur at home or on the way to the health facility—midwives should encourage women to attend antenatal clinics, where they would be told their due date and encouraged to seek care immediately labor sets in.

Health professionals should provide health education targeting family members, especially mothers-in-law, TBAs and older women, to encourage pregnant women to seek skilled delivery care.

Uneducated or less educated husbands should be sensitized about the importance of skilled delivery for informed decision making.

Ethnic groups that do not patronize health facility or skilled delivery services should be educated on the need for skilled care at birth.

## Competing interests

The author(s) declare that they have no competing interest.

## Authors’ contributions

ES conceived and designed the study. ES performed the data analysis, interpreted the results and wrote the manuscript. HVD and LM contributed to the planning and supervision of all parts of the study and the methodology and writing the manuscript. JB, KYA and SM contributed to the planning of the study and the revision of the manuscript. All authors read and approved the final version of the manuscript.

## Pre-publication history

The pre-publication history for this paper can be accessed here:

http://www.biomedcentral.com/1471-2458/14/344/prepub
